# The Relationship Between the Systemic Immune Inflammatory Index and Atrial Fibrillation in Heart Failure with Preserved Ejection Fraction

**DOI:** 10.5152/eurasianjmed.2026.261589

**Published:** 2026-08-11

**Authors:** Bihter Şentürk, Mehmet Kış, Tuğçe Çöllüoğlu, Hüseyin Dursun, Çisem Oktay, Firdevs Aysenur Ekizler, Mehmet Birhan Yılmaz

**Affiliations:** 1 Department of Cardiology, Dokuz Eylül University Faculty of Medicine, İzmir, Türkiye; 2 Department of Cardiology, Bilkent City Hospital, Ankara, Türkiye

**Keywords:** Atrial fibrillation, biomarkers, heart failure, inflammation, preserved ejection fraction, prognosis, systemic immune-inflammation index

## Abstract

**Background:**

Immunopathological dysregulation has been implicated as a unifying substrate for both atrial fibrillation (AF) and heart failure with preserved ejection fraction (HFpEF), 2 conditions that cluster with disproportionate frequency in clinical cardiology practice. Systemic immune-inflammation index (SII)—a hematological index derived from the ratio of platelet–neutrophil product to lymphocyte count—has garnered increasing attention as a low-burden inflammatory surrogate. The capacity to discriminate AF from sinus rhythm was investigated in an outpatient cohort with HFpEF.

**Methods:**

A retrospective analysis was conducted on 207 HFpEF outpatients (January 2024-January 2025) stratified by resting electrocardiogram rhythm: sinus rhythm (group 1, n = 105) vs. AF (group 2, n = 102). Independent AF predictors were identified through multivariate binary logistic regression.

**Results:**

Four variables independently predicted AF on multivariate analysis: SII (*P* = .04), age (*P* = .010), left atrial diameter (*P* < .001), and systolic pulmonary artery pressure (*P* < .001). At an optimal SII threshold of 584.64, sensitivity reached 79.4% and specificity 59% for AF prediction (AUC = 0.727, 95% CI = 0.658-0.796, *P* < .001).

**Conclusion:**

Systemic immune-inflammation index constitutes a cost-neutral, universally derivable hematological parameter with meaningful clinical utility for AF risk stratification within the HFpEF phenotype. Patients harboring pronounced SII elevation warrant heightened arrhythmia vigilance through serial 12-lead recordings and extended ambulatory electrocardiographic monitoring—should be considered in HFpEF patients with markedly elevated SII to facilitate timely AF detection.

**Main Points**

- Computed solely from 3 parameters of the routine complete blood count, SII demonstrated an independent association with atrial fibrillation (AF) occurrence in a well-characterized heart failure with preserved ejection fraction (HFpEF) cohort, without any additional diagnostic expenditure.
- Advancing age, left atrial dilation, and elevated pulmonary arterial systolic pressure each emerged as independent arrhythmogenic determinants within the same multivariable model.
- Receiver operating characteristic–derived thresholding identified an SII value of 584.64 as an operationally useful discriminant between AF and sinus rhythm, with clinically acceptable sensitivity and specificity characteristics.
- Stratifying HFpEF patients by SII magnitude could guide the allocation of extended electrocardiographic monitoring resources, prioritizing detection efforts toward those at highest arrhythmic risk.

## Introduction

Within the heart failure with preserved ejection fraction (HFpEF) population, atrial fibrillation (AF) represents one of the most diagnostically and prognostically consequential comorbidities, documented in one-third or more of affected individuals across contemporary registries.^1^^,^^2^ The superimposition of AF upon HFpEF substantially amplifies the hazard of decompensation-driven hospitalization, cardiovascular mortality, and embolic cerebrovascular events.^3^ Beyond left-sided consequences, AF independently degrades right ventricular and right atrial performance in HFpEF, an effect that persists irrespective of concomitant pulmonary hemodynamic derangements.^4^^,^^5^ The 2 conditions converge upon a shared cardiometabolic risk substrate, encompassing systemic hypertension (HT), glucose dysregulation, epicardial coronary disease, adiposity, sleep-disordered breathing, and tobacco exposure.^6^ Beyond this comorbidity clustering, accumulating mechanistic evidence positions sterile immune activation as a unifying upstream driver, with cytokine-orchestrated structural remodeling of both atrial and ventricular myocardium constituting the molecular substrate common to HFpEF and AF. Fibroinflammatory atrial remodeling and cardiomyocyte stiffening thus emerge as the cardinal pathophysiological bridge linking the 2 entities.^7^^-^^11^

In the HFpEF milieu, chronically elevated left ventricular end-diastolic pressures impose mechanical stress upon the posterior left atrial wall, engendering progressive atrial dilatation and fibrosis; superimposed inflammatory remodeling impairs intercellular electrochemical coupling, culminating in heterogeneous conduction that facilitates re-entrant circuit formation.^12^ Histopathological interrogation of surgically excised atrial appendage specimens has revealed leukocytic infiltrates, macrophage accumulation, and collagen deposition in AF patients, furnishing direct morphological corroboration of an inflammatory tissue phenotype.^13^ Experimental rodent models recapitulating aged HFpEF physiology have demonstrated that senescence-associated atrial transcriptomic reprogramming—characterized by pro-inflammatory cytokine upregulation and oxidative stress—confers enhanced inducibility of sustained atrial arrhythmias.^14^ The pro-inflammatory milieu further upregulates tissue factor expression and platelet activation cascades, compounding the intrinsic thromboembolic propensity of AF and potentially contributing to the disproportionate stroke burden observed when both conditions coincide.^15^

The systemic immune inflammatory index (SII) is a hematological composite encapsulating innate immune activation (neutrophilia, thrombocytosis) and adaptive immune attenuation (lymphopenia) within a single arithmetic construct—(platelet count × neutrophil count)/lymphocyte count—offering a parsimonious yet informationally rich characterization of systemic inflammatory state from a routine complete blood count (CBC) specimen.^16^ Its prognostic validity has been prospectively validated across an expanding range of cardiovascular phenotypes, including hypertensive cardiomyopathy, stable coronary artery disease (CAD), acute coronary syndromes, and heart failure with reduced ejection fraction.^17^^-^^20^ Notwithstanding this expanding evidence base, its discriminatory performance specifically within the HFpEF–AF intersection had not been examined prior to the present investigation, which was therefore designed to address this uncharacterized clinical domain.

## Material and Methods

**Figure 1.**
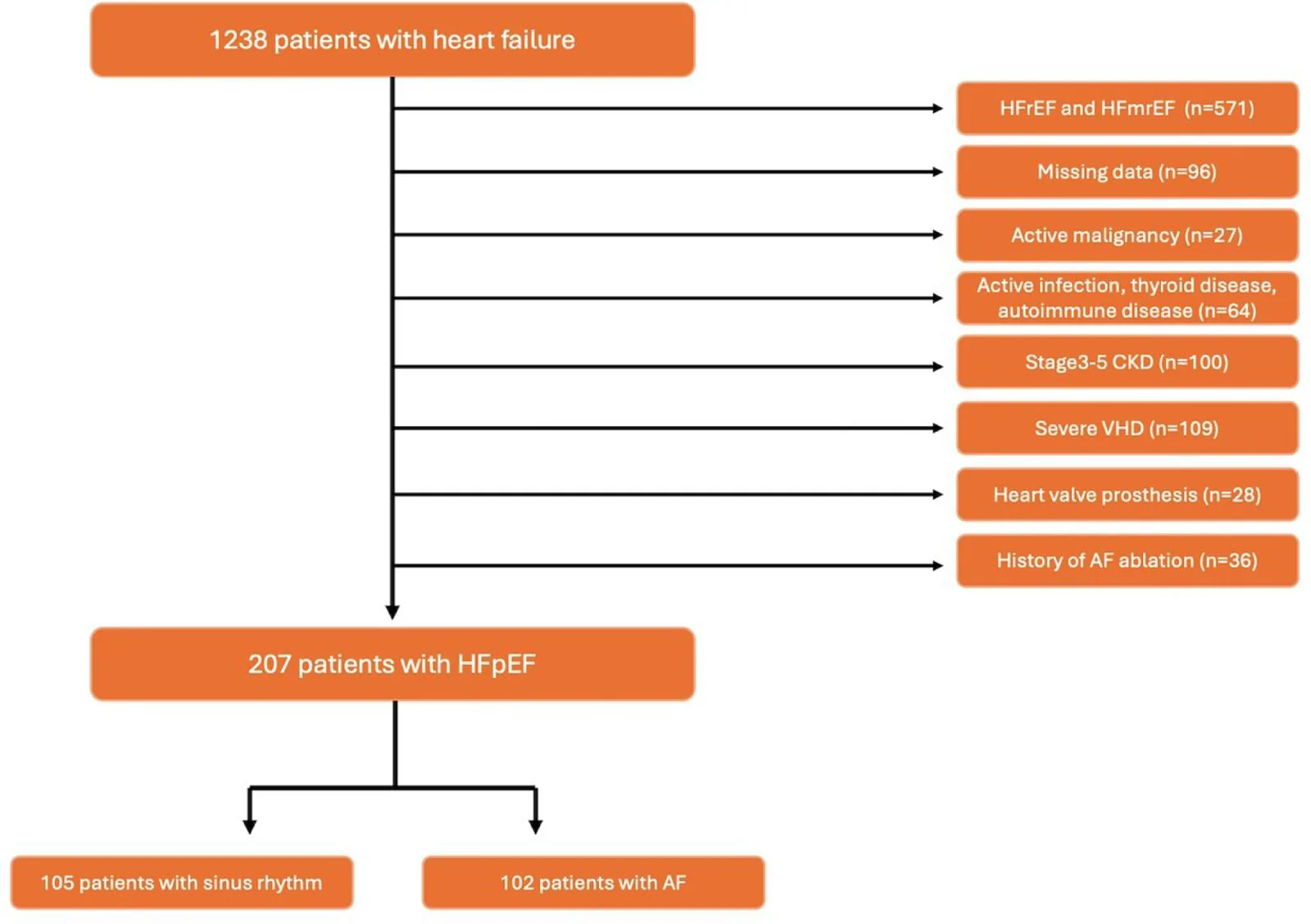
Flow-chart of the study population. AF, atrial fibrillation; CKD, chronic kidney disease; HFmEF, heart failure with mildly reduced ejection fraction; HFpEF, heart failure with preserved ejection fraction; HFrEF, heart failure with reduced ejection fraction; VHD, valvular heart disease.

Medical records of HFpEF patients attending the outpatient cardiology service between January 2024 and January 2025 were reviewed in a retrospective fashion. Heart failure with preserved ejection fraction diagnosis required fulfillment of contemporary guideline criteria.^21^ Exclusion criteria were: records insufficient for analysis, active or suspected malignancy, thyroid pathology, documented acute infection, prior ablation for AF, hemodynamically important native valve disease or prosthetic valve replacement, and pre-existing hematological, autoimmune, inflammatory, hepatic, or advanced renal disease (stages 3-5). After applying these criteria, 207 patients remained and were categorized by index electrocardiogram (ECG) into group 1 (n = 105, sinus rhythm) and group 2 (n = 102, AF) (Figure 1).

Using values extracted from routine CBC data in the institutional records, SII was computed as follows: SII = (platelet count × neutrophil count) / Lymphocyte count.^16^ The CBC measurements were drawn from results documented during the index outpatient encounter.

This study was a retrospective single-center observational analysis. Institutional ethics committee approval was secured from Dokuz Eylül University (Approval No: 2025/09-20, Date: March 12, 2025) Individual patient consent was not required owing to the retrospective nature of data retrieval. All aspects of the study conformed to the Declaration of Helsinki.

ChatGPT was employed as a language-editing aid during the preparation of this manuscript. 

### Statistical Analysis

Data processing and statistical testing were carried out in IBM SPSS Statistics, version 25.0 (IBM Corp., Armonk, NY). Normality was evaluated via the Kolmogorov–Smirnov test. Variables conforming to a Gaussian distribution are summarized as mean ± standard deviation; between-group comparisons employed Student’s *t*-test. Skewed variables are summarized as medians with interquartile ranges (Q1-Q3) and compared by the Mann–Whitney *U*-test. Categorical data are reported as counts and proportions; group differences were tested by Pearson’s chi-square or Fisher’s exact test as appropriate. To identify independent determinants of AF, binary logistic regression was performed sequentially in univariate then multivariate steps; variables achieving *P* ≤ .05 on univariate screening entered the multivariate model. The optimal SII discriminative threshold was established by receiver operating characteristic (ROC) analysis. Statistical significance required *P* ≤ .05.

## Results

Overall mean age was 62.68 years; group 2 patients were significantly older (*P* = .048). Women comprised roughly half the study population (51.2%) and were proportionally more represented in group 2 (*P* = .031). The prevalences of HT (52.7%), diabetes mellitus (DM) (41.1%), and CAD (35.3%) did not differ meaningfully between the 2 groups (*P* = .843, .752, and .555, respectively). Left ventricular ejection fraction, end-diastolic diameter, and end-systolic diameter were statistically equivalent across groups (*P* = .514, .446, and .908, respectively), whereas mean left atrial diameter (LAD) and mean systolic pulmonary artery pressure (SPAP) were each significantly higher in group 2 (*P* < .001 for both) (Table 1).

Routine biochemical parameters—including glucose, creatinine, sodium, potassium, aspartate aminotransferase, alanine aminotransferase, and lipid fractions—did not differ significantly between groups (all *P* > .05). Group 2 showed a significantly lower lymphocyte count (*P* < .001) alongside significantly higher brain natriuretic peptide, neutrophil count, platelet count, neutrophil-to-lymphocyte ratio, and SII values (*P* < .001, *P* = .002, *P* < .001, *P* < .001, respectively) (Table 2).

**Figure 2.**
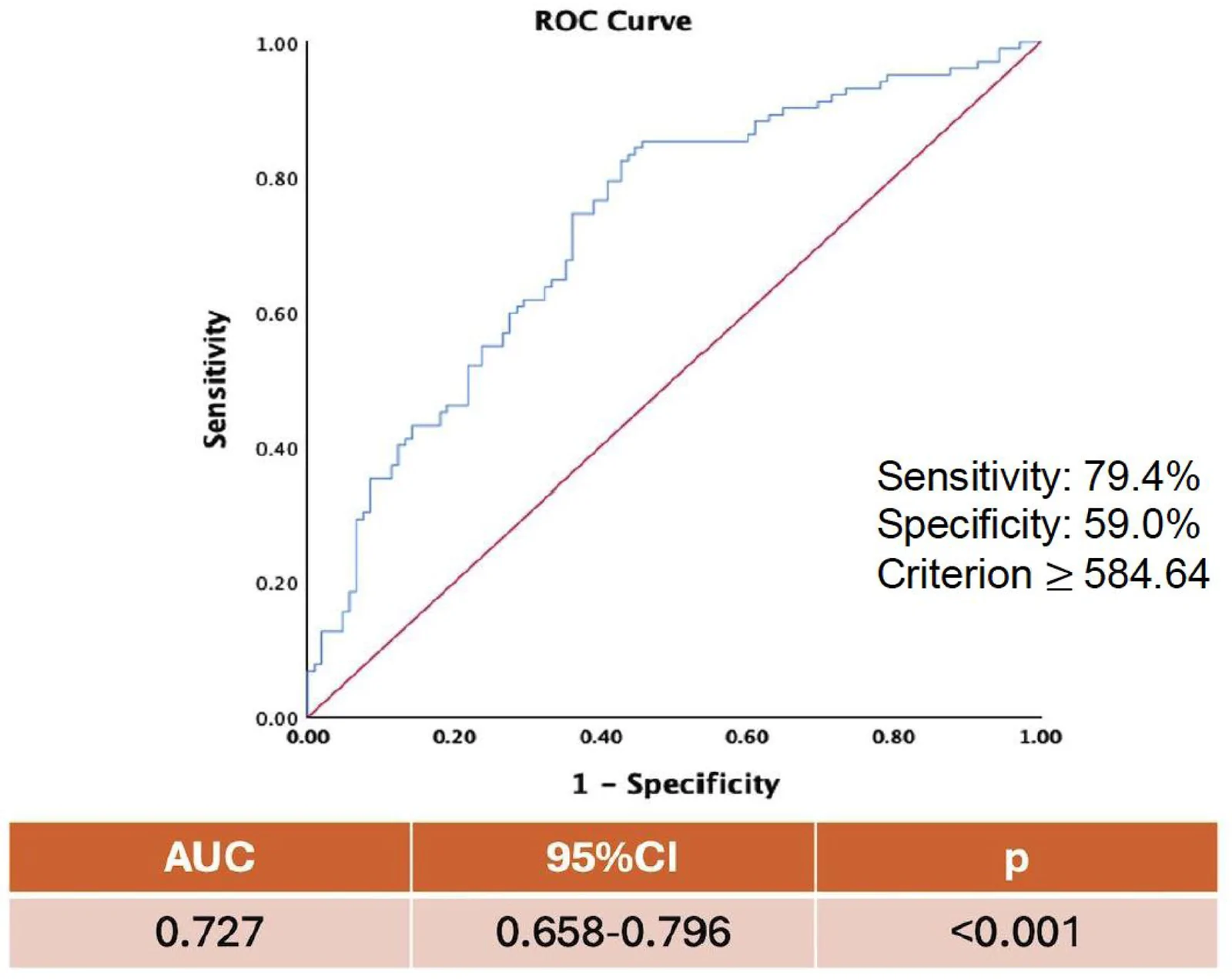
Receiver operating characteristic curves of systemic immune inflammatory index for predicting atrial fibrillation. AUC, area under the curve; ROC, receiver operating characteristic.

On multivariate logistic regression, SII (*P* = .04), age (*P* = .010), LAD (*P* < .001), and SPAP (*P* < .001) each independently predicted AF (Table 3). The optimal SII threshold by ROC analysis was 584.64, at which sensitivity was 79.4% and specificity was 59% (AUC = 0.727, 95% CI = 0.658-0.796, *P* < .001) (Figure 2).

## Discussion

Heart failure with preserved ejection fraction and AF engage in a bidirectional pathophysiological symbiosis that transcends simple comorbidity. Rapid ventricular response during AF imposes acute diastolic loading conditions that unmask occult ventricular stiffness, while the chronically elevated left atrial pressures intrinsic to HFpEF progressively distend and electromechanically remodel atrial myocardium, fostering the fibrotic heterogeneity and conduction slowing that sustain re-entrant arrhythmogenesis.^1^^,^^12^ The adverse prognostic synergism of this comorbid pairing—conferring incremental risk beyond that attributable to either condition alone—underscores the imperative for systematic arrhythmia surveillance within the HFpEF care pathway.^3^

A paradigm shift has repositioned HFpEF as an inflammatory cardiomyopathy, in which microvascular endothelial dysfunction driven by systemic comorbidities initiates a cytokine-mediated cascade of myocardial injury.^22^ Downstream consequences of this inflammatory cascade include titin hypophosphorylation, reactive oxygen species-mediated cardiomyocyte hypertrophy, and lysyl oxidase-mediated collagen cross-linking—collectively augmenting passive myocardial stiffness and impeding ventricular relaxation kinetics.^23^ Concomitant atrial exposure to circulating pro-inflammatory cytokines, including interleukin-6, interleukin-1β, and transforming growth factor-β, drives fibroblast transdifferentiation, extracellular matrix deposition, and gap junction remodeling within the atrial wall, collectively generating the electrophysiological substrate that sustains AF^24^ Concomitant downregulation of connexin-40 and -43 expression by nuclear factor-κB-driven inflammatory signaling impairs rapid intercellular conduction, predisposing to the wavelength shortening and conduction heterogeneity prerequisite for re-entrant AF maintenance.^25^ Epidemiological data from prospective community cohorts corroborate this mechanistic framework, demonstrating that circulating high-sensitivity C-reactive protein and soluble tumor necrosis factor-α receptor concentrations each independently forecast incident AF and diastolic dysfunction, with additive elevation when both conditions manifest concurrently.^26^^,^^27^

Visceral and epicardial adiposity superimpose an additional inflammatory burden upon this shared pathogenic framework. Hypertrophied epicardial adipocytes elaborate resistin, leptin, and pro-inflammatory interleukins in a paracrine fashion directly targeting subepicardial myocardium and the adjacent pulmonary veins, instigating microvascular rarefaction, atrial wall fibrosis, and electrophysiological remodeling.^28^ Incremental adipose accumulation amplifies the paracrine adipokine gradient impinging on atrial cardiomyocytes, simultaneously prolonging local action potential duration, reducing conduction velocity, and augmenting diastolic filling pressures—mechanistic correlates that explain the disproportionate prevalence of concomitant AF and HFpEF among individuals with high body mass.^29^

Aptamer-based proteomic profiling of patients with concomitant HF and AF has revealed phenotype-specific molecular fingerprints: HFrEF is characterized by predominant activation of the neurohormonal and cardiorenal axes, whereas HFpEF exhibits disproportionate upregulation of adipose tissue-derived proteins and adiponectin pathway intermediates, corroborating the central role of metabolic inflammation in HFpEF pathobiology.^30^ In a metabolically dysregulated hepatic steatosis phenotype of metabolic-associated steatotic liver disease—marked by adipose insulin resistance, ectopic lipid deposition, and dysregulated adipokine secretion—reprograms epicardial adipose tissue toward a pro-fibrotic, pro-arrhythmic secretory phenotype, creating concurrent susceptibility to both AF perpetuation and HFpEF progression.^31^

Interventional pharmacological evidence substantiates the therapeutic relevance of this axis. Hydroxymethylglutaryl-CoA reductase inhibitors and anti-cytokine biologics—attenuate atrial myopathic changes and appear to reduce the incidence of both conditions. Compounds capable of remodeling the epicardial adipose inflammatory microenvironment—sodium-glucose cotransporter-2 inhibitors, glucagon-like peptide-1 receptor agonists, and thiazolidinediones—represent mechanistically coherent candidates for modifying the shared adipoinflammatory driver of concomitant AF and HFpEF.^32^ This convergent pharmacological evidence positions immune-inflammatory remodeling as a tractable therapeutic target whose modulation may simultaneously attenuate arrhythmic burden and diastolic dysfunction in this high-risk comorbid phenotype.

The arrhythmogenic relevance of SII has been validated across heterogeneous cardiovascular disease contexts. In unselected ischemic stroke registries, SII discriminated patients with underlying AF from those in sinus rhythm with independent predictive significance—a finding with direct implications for secondary stroke prevention.^33^ Pooled analysis of 8 perioperative cohorts established a statistically robust and clinically significant association between preoperative SII magnitude and incident postoperative atrial arrhythmia.^34^ Within ambulatory rhythm monitoring registries, graded SII elevation correlated with paroxysmal AF occurrence, suggesting its utility as a pre-screening biomarker preceding more resource-intensive monitoring strategies.^35^

In the acute coronary setting, admission SII independently predicted incident peri-infarction AF among patients with ST-elevation myocardial infarction, reinforcing inflammation as a mechanistic driver of arrhythmia vulnerability in the acutely ischemic myocardium.^36^ Among patients with DM undergoing pulmonary vein isolation, baseline SII independently stratified the risk of post-ablation arrhythmia recurrence, with implications for patient selection and post-procedural surveillance intensity.^37^ In head-to-head comparisons of inflammatory indices, SII outperformed the conventional neutrophil-to-lymphocyte ratio as a predictor of electrical cardioversion failure and early arrhythmia reinduction.^38^ SII also exhibited superior discrimination for post-cryoablation AF recurrence relative to other tested inflammatory composites, suggesting mechanistic specificity beyond generic immune activation.^39^

Notably, SII demonstrates a dose–response relationship with arrhythmia chronification: values escalate progressively across the paroxysmal, persistent, and permanent AF spectrum, and remain consistently elevated relative to sinus rhythm controls, suggesting that cumulative inflammatory burden accelerates arrhythmia progression.^40^ The present investigation situates the HFpEF–AF comorbidity within the established SII arrhythmia-biomarker framework, demonstrating that SII retains independent predictive validity within the immune-metabolic milieu of diastolic heart failure, consistent with the growing literature on SII as an arrhythmia biomarker. The mechanistic rationale linking systemic inflammation to both HFpEF and AF lends further biological plausibility to this finding and supports SII as a practical tool for identifying high-risk patients.

Several methodological constraints warrant acknowledgment. First, the non-randomized retrospective design. Second, arrhythmia classification was predicated exclusively on a single resting 12-lead electrocardiogram, an approach that systematically underestimates true AF prevalence; patients phenotyped as sinus rhythm may harbor temporally discrete paroxysmal episodes detectable only through implantable loop recorder interrogation or extended Holter surveillance. Third, anthropometric body composition data, including body mass index, were not systematically retrievable from the institutional records; given that visceral adiposity independently drives both atrial arrhythmogenesis and diastolic ventricular dysfunction,^29^ subsequent investigations should explicitly incorporate adipometric parameters as covariates to disentangle SII-specific inflammatory risk from adiposity-mediated confounding. Finally, the constrained sample size limits effect estimate precision; external validation in geographically and ethnically diverse prospective cohorts is therefore essential before definitive clinical implementation.

In summary, as an SII—an index computable from standard CBC data at zero incremental cost—offers meaningful clinical utility for augmenting AF screening and shaping a risk-stratified surveillance approach in HFpEF patients. Clinicians encountering HFpEF patients with SII values above the identified threshold should consider intensifying rhythm monitoring through repeated ECGs and prolonged ambulatory recording. Prospective trials are warranted to determine whether anti-inflammatory therapies can reduce AF incidence among HFpEF patients with high SII values.

## Figures and Tables

**Table 1.** Demographic Characteristics, Comorbidities, and Echocardiographic Findings of the Patients

Variable	Group 1: SR (n = 105)	Group 2: AF (n = 102)	*P*
Age (years)	60.90 ± 13.43	64.51 ± 12.70	.048*
Female gender, n (%)	46 (43.4)	60 (56.6)	.031*
HT, n (%)	56 (53.3)	53 (52.0)	.843
DM, n (%)	42 (40.0)	43 (42.2)	.752
CAD, n (%)	35 (33.3)	38 (37.3)	.555
LVEF (%)	57.53 ± 5.40	57.06 ± 5.03	.514
LVEDD (mm)	47.39 ± 4.70	47.88 ± 4.56	.446
LVESD (mm)	29.91 ± 4.86	30.00 ± 4.57	.908
LAD (mm)	35.57 ± 4.36	44.77 ± 5.24	<.001*
SPAP (mmHg)	30.77 ± 4.40	35.05 ± 4.13	<.001*

AF, atrial fibrillation; CAD, coronary artery disease; DM, diabetes mellitus; HT, hypertension; LAD, left atrial diameter; LVEDD, left ventricular end-diastolic diameter; LVEF, left ventricular ejection fraction; LVESD, left ventricular end-systolic diameter; SPAP, systolic pulmonary artery pressure; SR, sinus rhythm.

^*^P < .05.

**Table 2.** Laboratory Findings of the Patients

Parameter	Group 1: SR (n = 105)	Group 2: AF (n = 102)	*P*
Glucose (mg/dL)	104 (92.5-127.5)	108 (92.0-142.2)	.390
Creatinine (mg/dL)	1.15 ± 0.67	1.11 ± 0.51	.658
Sodium (mmol/L)	138.36 ± 2.66	138.97 ± 2.66	.102
Potassium (mmol/L)	4.31 ± 0.50	4.35 ± 0.74	.606
BNP (ng/dL)	209 (156-456)	766 (361-1084)	<.001*
AST (U/L)	20 (16-23)	20 (15.7-24)	.704
ALT (U/L)	20 (15-24)	18 (13-24)	.349
Hemoglobin (g/dL)	13.43 ± 1.99	13.23 ± 2.08	.479
WBC (10^3^/μL)	9.67 (7.76-12.44)	9.85 (7.90-12.49)	.712
Platelets (10^3^/μL)	239.1 ± 48.6	263.6 ± 55.5	.001*
Neutrophils (10^3^/μL)	5.90 (4.58-7.36)	7.61 (5.20-9.32)	.002*
Lymphocytes (10^3^/μL)	2.25 (1.83-3.20)	1.90 (1.29-2.42)	<.001*
NLR	2.31 (1.71-3.86)	3.62 (2.43-6.29)	<.001*
SII	524.31 (364.87-889.83)	939.58 (636.07-1510.36)	<.001*
Total cholesterol (mg/dL)	196 (153-218)	180 (153-216)	.419
Triglycerides (mg/dL)	151 (110-202)	154 (109-190)	.413
HDL-C (mg/dL)	38.5 (34-48)	41.2 (35-48.5)	.158
LDL-C (mg/dL)	112 (80.3-140)	110 (80.3-132.8)	.874

AF, atrial fibrillation; ALT, alanine aminotransferase; AST, aspartate aminotransferase; BNP, brain natriuretic peptide; HDL-C, high-density lipoprotein cholesterol; LDL-C, low-density lipoprotein cholesterol; NLR, neutrophil-to-lymphocyte ratio; SII, systemic immune-inflammation index; SR, sinus rhythm; WBC, white blood cell count.

*P *< .05.

**Table 3.** Univariate and Multivariate Logistic Regression Analyses for Predicting Atrial Fibrillation

Variables	Univariate Logistic Regression			Multivariate Logistic Regression		
	OR	95% CI	*P*	OR	95% CI	*P*
Age (years)	1.021	1.000-1.040	.050*	1.047	1.011-1.084	.010*
Female gender	1.832	1.056-3.180	.031*	1.193	0.410-3.477	.746
Left atrial diameter (mm)	1.541	1.373-1.730	<.001*	1.612	1.387-1.874	<.001*
SPAP (mmHg)	1.257	1.165-1.356	<.001*	1.277	1.135-1.438	<.001*
BNP (ng/dL)	1.001	1.001-1.002	<.001*	1.000	0.999-1.001	.947
SII	1.001	1.000-1.001	<.001*	1.001	1.000-1.001	.004*

BNP, brain natriuretic peptide; CI, confidence interval; LVEF, left ventricular ejection fraction; OR, odds ratio; SII, systemic immune-inflammation index; SPAP, systolic pulmonary artery pressure.

*P < .05.

## Data Availability

The data that support the findings of this study are available on request from the corresponding author.
